# Supplementary material for: RPA, an Accurate and Fast Method for the Computation of Static Nonlinear Optical Properties
Source: J Chem Theory Comput. 2023 Sep 11;19(18):6062–9. doi: 10.1021/acs.jctc.3c00674 (PMC10861135; doi:10.1021/acs.jctc.3c00674)
Supplement: Supplementary file 1 — ct3c00674_si_001.pdf [file ct3c00674_si_001.pdf]

Supporting information for: RPA, an accurate and fast method for the computation of static non-linear optical properties

Pau Besalú-Sala,[†,‡] Fabien Bruneval,[¶] Ángel José Pérez-Jiménez,[§] Juan Carlos Sancho-García,[§] and Mauricio Rodríguez-Mayorga[*,§]

†*Department of Chemistry and Pharmaceutical Sciences, Amsterdam Institute for Molecular and Life Sciences (AIMMS), Vrije Universiteit Amsterdam, De Boelelaan 1083, 1081 HV Amsterdam, The Netherlands*

‡*Institut de Química Computacional i Catàlisi and Departament de Química, Universitat de Girona, 17003 Girona, Spain*

¶*Université Paris-Saclay, CEA, Service de recherche en Corrosion et Comportement des Matériaux, SRMP, 91191 Gif-sur-Yvette, France*

§*Department of Physical Chemistry, University of Alicante, E-03080 Alicante, Spain*

E-mail: marm3.14@gmail.com

Computational details

We have implemented in `MOLGW` code[1,2] the homogeneous electric fields $\mathbf{F}$, needed for the computation of the numerical (hyper)-polarizabilities in Eq. (1) in the main text. In this work, we have used finite differences computed with an *in-house* program that utilizes the Rutishauser–Romberg scheme[3,4] to reduce the numerical errors (see Refs. 5 and 6 for more

details). Furthermore, the KS DFT double-hybrid functional approximations tested in this work (i.e. B2PLYP, PBE0-DH, and PBE-QIDH) have been introduced in `MOLGW` code[7] In addition, the density-fitting technique has been employed with the aug-cc-pVDZ-RI auxiliary basis set[8,9] in all calculations. Finally, the RPA correlation energy given in Eq. (4) in the main text has been computed using the density fitting technique and a Gauss–Legendre quadrature with 40 imaginary frequencies, which has proven to ensure high accuracy in the integration with errors lower than 10^{-6} a.u.

Tα-LC-BLYP construction

The construction of the tuning scheme used by the optimally tuned (OT) range-separated functional called Tα-LC-BLYP is described in this section.

In the original work, the pristine functional LC-BLYP was chosen for the tuning process, because it is the (non-OT) DFA that presented the lower relative error compared to CCSD(T); thus, serving as a good starting point.[6] The tuning scheme was derived for the purpose to improve the results of LC-BLYP for the calculation of the second hyperpolarizability of the molecules in the so-called γ-NLO set, which is also employed in this work.

The tuning process requires the computation of the l_α indicator

$$l_\alpha = \log\left(\frac{\alpha_{zz}}{N}\right), \tag{1}$$

where the α_{zz} values are the ones obtained using the pristine LC-BLYP functional, and N is the number of electrons. Since there exists a quadratic correlation between l_α and the range-separation parameter (ω), this relationship is used in LC-BLYP to improve the predicted values w.r.t. the CCSD(T) reference ones. Then, the fact that α and γ are both even derivatives of the electronic energy is exploited using the number of electrons as a normalization factor to improve the prediction of γ. The logarithm in Eq. 1 is used to shrink down the multiple possible values that $\frac{\alpha}{N}$ can take to the range of values that ω

usually presents (i.e. between 0 and 1).

The quadratic correlation determining the ω used is the following,

$$\omega_{T\alpha} = 0.6269 l_\alpha^2 - 0.4556 l_\alpha + 0.3791, \tag{2}$$

which shows a $r^2 = 0.77$.

The physical reason behind the success of Eq. 2 to compute second hyperpolarizabilities, as well as an assessment of its very good performance for computing lower-order properties can be found in the original work.[6]

α_{zz}/n against n

Here we focus on the quality of the results for α_{zz}/n when the number of monomers is increased (as we did in the main text for the analysis of γ_{zzzz}/n). To that end, we have plotted in Fig. 1 the α_{zz}/n values obtained with CCSD(T), Tα-LC-BLYP, LC-BLYP, PBE-QIDH, and RPA@PBEh(0.85) against the number of monomers (n) for polymeric systems. From Fig. 1 we observe that RPA@PBEh(0.85) is the best approximation for Hydrogen chains, PA, and PDA. And, it is the second-best approximation for the PMI polymers. This method is able to reproduce the increase in the polarizability upon the increase of the monomer units. These results are in line with the fact that the lowest MEAN% and MAX%, for the predicted α_{zz} values, are obtained with the RPA@PBEh(0.85) approximation. Also, we want to point out that all the functionals considered here overestimate the reference CCSD(T) values, especially the PBE-QIDH one when n is increased.

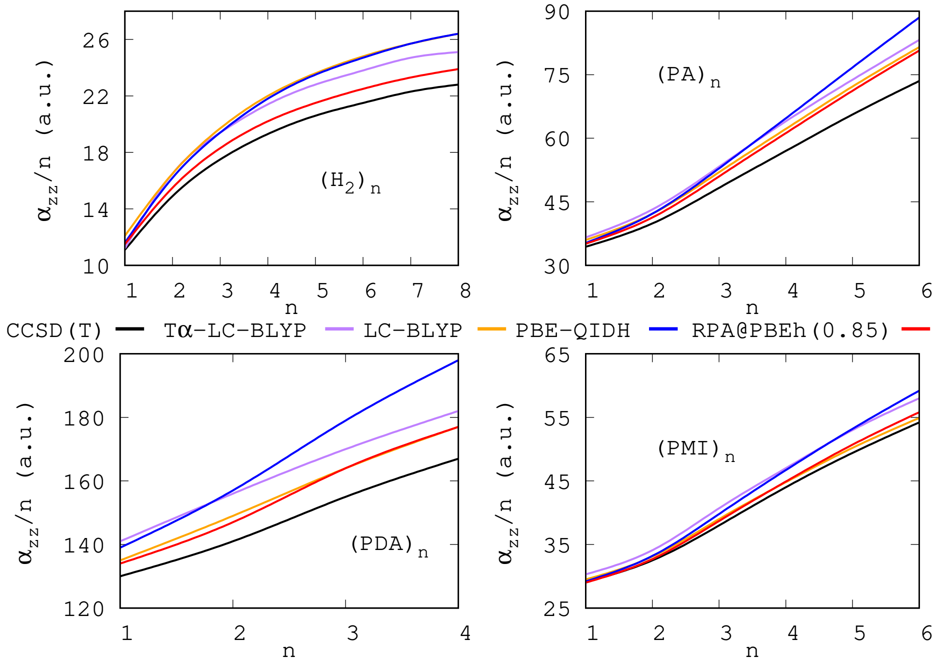

Figure 1: α_{zz} values per number of monomers against the number of monomers (n) for Hydrogen chains, PA, PDA, and PMI.

Further analysis of the SNLOPs. Separation by magnitude.

Since the relative indicators %MEAN and %MAX might hinder the usual density functional approximations and tend to amplify the errors on SNLOPs of larger systems, we have adopted an alternative evaluation approach. Initially, we organized the entire set of molecules based on their CCSD(T) property. Next, we divided the data into three subsets of equal size, categorized according to the size of the systems. Within each subset, we independently computed the %MEAN and %MAE for all the studied SNLOPs.

The impact of this modified approach on %MEAN and %MAX, in comparison to considering all molecules as described in the main text, has been detailed in Table 1. The list of molecules that belongs to each subset (for each property) is given in the Excel sheet added as supplementary material.

Table 1: Analysis of %MEAN and %MAX by size of the property. The letter in parenthesis after the indicator establishes which subset is being analyzed, with the code (A) all the systems, (B) the smallest-property subset, (C) the medium-property subset, and (D) the largest-property subset.

		Tα-LC-BLYP	LC-BLYP	PBE-QIDH	RPA@PBEh(0.85)
μ_z	%MEAN(A)	5.03	29.89	13.88	13.67
	%MAX(A)	67.11	174.32	46.36	52.61
	%MEAN(B)	26.73	26.54	14.29	14.55
	%MAX(B)	67.11	48.75	27.39	52.61
	%MEAN(B)	13.73	16.42	13.04	12.44
	%MAX(B)	44.34	40.43	46.36	45.43
	%MEAN(D)	14.25	20.35	14.40	14.09
	%MAX(D)	20.37	25.90	20.03	19.05
α_{zz}	%MEAN(A)	5.66	4.56	5.45	3.07
	%MAX(A)	13.15	82.29	16.19	9.73
	%MEAN(B)	3.26	2.57	1.99	2.08
	%MAX(B)	10.4	10.9	8.45	4.11
	%MEAN(C)	4.62	4.04	3.01	2.43
	%MAX(C)	10.9	14.6	13.9	4.61
	%MEAN(D)	9.32	7.22	11.7	4.79
	%MAX(D)	13.2	16.2	20.4	9.73
β_{zzz}	%MEAN(A)	73.59	61.14	39.20	49.34
	%MAX(A)	433.48	413.13	258.14	121.06
	%MEAN(B)	134.60	106.70	68.65	44.86
	%MAX(B)	433.48	413.13	258.14	121.06
	%MEAN(C)	23.73	15.30	9.69	27.31
	%MAX(C)	58.67	50.63	27.06	97.32
	%MEAN(D)	60.19	61.48	39.25	81.14
	%MAX(D)	91.06	91.73	89.41	91.59
γ_{zzzz}	%MEAN(A)	5.92	19.44	19.13	13.90
	%MAX(A)	29.32	50.73	78.45	49.50
	%MEAN(B)	7.16	16.01	15.73	16.01
	%MAX(B)	29.32	50.73	57.30	49.50
	%MEAN(C)	5.56	24.59	12.06	15.83
	%MAX(C)	28.44	33.40	76.55	29.49
	%MEAN(D)	4.97	17.62	30.25	9.60
	%MAX(D)	12.86	33.58	78.46	30.96

While the general tendency is that for molecules bearing larger SNLOPs the errors of the DFAs are larger, there are few exceptions. For instance, the relative errors of Tα-LC-BLYP are larger for group (B) than for groups (C) or (D). Remarkably, RPA@PBEh(0.85) is able

to reduce the differences among the three subsets compared to other DFAs. For instance, for α_{zz} the errors are stable in the range 2.08-4.79 for all three sets (being set D the one with larger errors). However, LC-BLYP presents, for the same property, a wider range of errors, i.e. 2.57-24.8 being set D again the set with larger errors. Hence, let us remark that the %MEAN and %MAX errors obtained for γ_{zzzz} for the subset containing the large systems indicate that RPA@PBEh(0.85) is indeed a good approximation for predicting SNLOPs; being only outperformed by the Tα-LC-BLYP approximation (similar conclusions can be raised from the analysis of α_{zz}). Finally, let us comment that with the exception of the (D) subset in the γ_{zzzz} prediction, the %MEAN and %MAX values are in general reasonably accurate using the PBE-QIDH functional.

References

(1) Bruneval, F.; Rangel, T.; Hamed, S. M.; Shao, M.; Yang, C.; Neaton, J. B. Molgw 1: Many-body perturbation theory software for atoms, molecules, and clusters. *Comput. Phys. Commun.* **2016**, *208*, 149 – 161.

(2) MOLGW code. `http://www.molgw.org`, last accessed on 10/04/2023.

(3) Rutishauser, H. Ausdehnung des Rombergschen Prinzips. *Numer. Math.* **1963**, *5*, 48–54.

(4) Medveď, M.; Stachová, M.; Jacquemin, D.; André, J.-M.; Perpete, E. A. A generalized Romberg differentiation procedure for calculation of hyperpolarizabilities. *J. Mol. Struct. THEOCHEM* **2007**, *847*, 39–46.

(5) Naim, C.; Besalú-Sala, P.; Zaleśny, R.; Luis, J. M.; Castet, F.; Matito, E. Are Accelerated and Enhanced Wave Function Methods Accurate to Compute Static Linear and Nonlinear Optical Properties? *J. Chem. Theory Comput.* **2023**, *19*, 1753–1764.

(6) Besalú-Sala, P.; Sitkiewicz, S. P.; Salvador, P.; Matito, E.; Luis, J. M. A new tuned range-

separated density functional for the accurate calculation of second hyperpolarizabilities. *Phys. Chem. Chem. Phys.* **2020**, *22*, 11871–11880.

(7) The latest release of `MOLGW` code (version 3.2) incorporates these new features (download from `https://github.com/bruneval/molgw/releases/tag/v3.2`, last accessed on 05/06/2023).

(8) Hättig, C.; Hellweg, A.; Köhn, A. Distributed memory parallel implementation of energies and gradients for second-order Møller–Plesset perturbation theory with the resolution-of-the-identity approximation. *Phys. Chem. Chem. Phys.* **2006**, *8*, 1159–1169.

(9) Weigend, F.; Köhn, A.; Hättig, C. Efficient use of the correlation consistent basis sets in resolution of the identity MP2 calculations. *J. Chem. Phys.* **2002**, *116*, 3175–3183.
